# Herzschrittmacherinfektion bei fragilen Patienten

**DOI:** 10.1007/s00399-023-00940-9

**Published:** 2023-04-28

**Authors:** Ernesto Casorelli, Ilaria Pescatori, Gaetano Ruocco, Hendrik Bonnemeier, Ojan Assadian, Franco Bui

**Affiliations:** 1 Department for cardiology and intensive care medicine, Valdichiana Hospital, Località Nottola, Montepulciano, Italien; 2 Helios Klinik Cuxhaven, Altenwalder Chaussee 10, 27474 Cuxhaven, Deutschland; 3 Helios Klinik Wesermarsch, Mildred-Scheel-Straße 1, 26954 Nordenham, Deutschland; 4 Medizinische Fakultät, Christian-Albrechts-Universität, Christian-Albrechts-Platz 4, 24118 Kiel, Deutschland; 5 Landesklinikum Wiener Neustadt, Corvinusring 3–5, 2700 Wiener Neustadt, Österreich

**Keywords:** Herzrhythmusstörungen, Kardiale Implantate, Fistel, Sondenextraktion, Taurolidin, Cardiac arrhythmias, Cardiac implantable electronic device (CIED), Fistula, Lead extraction, Taurolidine

## Abstract

Die Komplikationen nach Schrittmacherimplantation sind vielfältig. Neben Dislokation der Elektroden, Twiddler-Syndrom, Fehlfunktion und Hämatombildung kann es zu Schrittmacherinfektionen kommen. Diese können in akute, subakute und späte Infektionen unterteilt werden. Sowohl der Zeitpunkt des Auftretens als auch der Infektionsweg spielen eine entscheidende Rolle. Die Folgen einer Schrittmacherinfektion sind verheerend. Zur leitliniengerechten Behandlung gehört die Entfernung aller implantierten Anteile des Schrittmachers (transvenöse und subkutane Anteile). Wird keine vollständige Entfernung vorgenommen, kommt es häufig erneut zur Infektion. Die offene Thoraxchirurgie zur Entfernung infizierter Schrittmacherkomponenten ist mittlerweile durch perkutane Extraktionsverfahren ersetzt worden. Die Sondenextraktion erfordert spezielles Gerät und Expertise, welche nicht immer vorhanden sind. Zudem ist sie nicht bei jedem Patienten durchführbar. Jedes Extraktionsverfahren ist mit einem geringen Risiko potenziell tödlich verlaufender Komplikationen verbunden (z. B. Einriss kardialer oder vaskulärer Strukturen, Hämatothorax oder Herztamponade). Aus genannten Gründen sollte die Durchführung solcher Verfahren spezialisierten Zentren mit entsprechender Ausrüstung und Erfahrung vorbehalten sein. In diesem Fallbericht werden die Schritte zur erfolgreichen Revision eines Herzschrittmachers bei einem fragilen Patienten ohne Möglichkeit der Extraktion erläutert, welcher sich mehr als 5 Jahre nach Generatorwechsel mit einer Fistel im Bereich der Generatortasche in unserer Ambulanz vorstellte.

## Abstract (en)

Complications associated with cardiac implantable electric devices (CIED) are manifold. They include lead dislocation, twiddler’s syndrome, device malfunction, haematoma formation and infection. Infections can be divided into acute, subacute and late infections. Both the time of onset and the route of infection play a crucial role. The consequences of a CIED infection are devastating. The most modern treatment methods include the removal of all implanted implants. If complete removal is not followed in the event of infection, there is a high rate of infection recurrence. Open thoracic surgery to remove infected CIED hardware has been replaced by percutaneous lead extraction procedures. Lead extraction requires specialised equipment and expertise and may not be readily available or feasible for some patients. Each extraction procedure is associated with a small risk of potentially fatal complications (e.g. cardiac avulsion, vascular avulsion, haemothorax and cardiac tamponade). For these reasons, the performance of such procedures should be limited to centres with adequate equipment and experience. Successful salvage of CIED systems with in situ sterilisation of contaminated hardware has been reported. In our case, we report the successful salvage of an exposed generator in a frail patient treated more than 5 years after the last generator replacement.

## Anamnese

**Figure 1:**
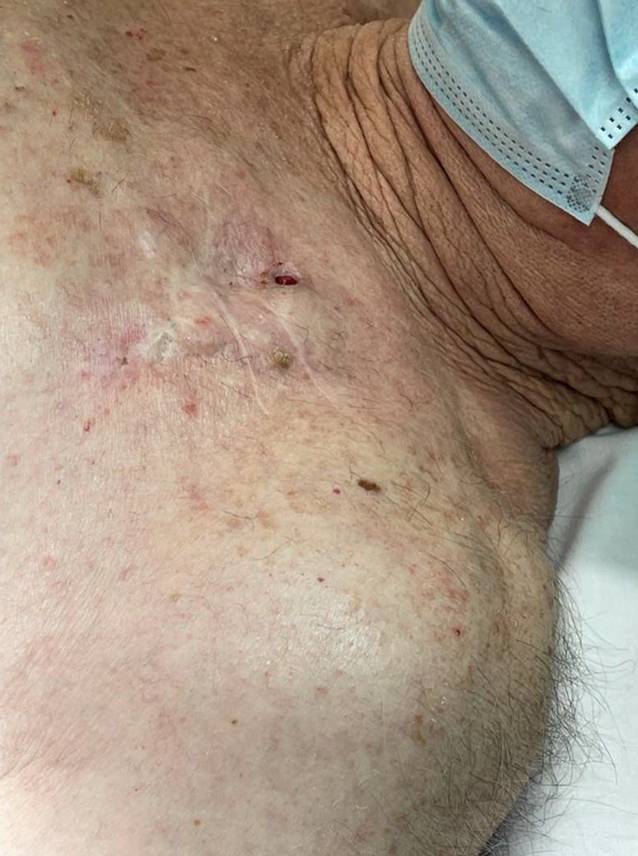

Ein 89-jähriger Patient wird mit einer Hauterosion im Bereich über einem implantierbaren Kardioverter-Defibrillator (ICD) in unserer Ambulanz vorstellig (Abb. 1). Der Patient hatte sein erstes Implantat, einen frequenzadaptiven Zweikammer-ICD (DDDR-ICD) bereits im Alter von 69 Jahren erhalten, bei bekannter ischämischer Kardiomyopathie (ICMP) mit mittelgradig eingeschränkter linksventrikulärer Ejektionsfraktion (LVEF: 40 %). Der letzte Generatorwechsel war zum Zeitpunkt der Vorstellung in der Ambulanz 5 Jahre vorher, mit der Indikation eines elektiven Wechsel bei Batterieerschöpfung (ERI), erfolgt. Der klinische Verlauf war bis dato unauffällig gewesen. Drei Jahre zuvor stellte sich das Gerät im Rahmen des „Mode Switch“ bei permanentem Vorhofflimmern auf eine ventrikuläre Interventionsweise um (VVI-R).

## Untersuchung

Bei der körperlichen Untersuchung zeigte sich ein klares Exsudat im Bereich der Hauterosion, welche sich bei genauerer Betrachtung als Fistelung mit Zugang zur fibrösen Aggregattasche darstellte. Die umliegende Haut imponierte nicht entzündet, der Patient zeigte sich, unter optimaler medikamentöser Therapie, kardial kompensiert und ohne direkte oder indirekte Anzeichen einer systemischen Entzündung oder Infektion.

## Diagnostik

In der Laborkonstellation imponierten neben einer Niereninsuffizienz im Stadium der dekompensierten Retention keinerlei Hinweise für eine systemische Entzündung oder ein Infektgeschehen. Mehrzeitig an verschiedenen Punktionsstellen entnommene Blutkulturen zeigten ebenso wie Gewebeproben und Abstriche aus dem Bereich der Fistel keinerlei Pathogenwachstum. Im Bereich der Aggregattasche zeigte sich im Rahmen der sonographischen Untersuchung (Linearschallkopf) kein Flüssigkeitsverhalt. Auch die transthorakale (TTE) und transösophageale (TOE) Echokardiographie blieben ohne Nachweis endokarditissuspekter Anhaftungen an den transvenösen Anteilen der Sonden oder an endokardialen Strukturen. In der Röntgen-Thorax-Untersuchung waren keine Infiltrate oder Stauungszeichen bei global vergrößertem Herzschatten sichtbar. Die Recessi imponierten frei.

## Therapie und Verlauf

In der Annahme eines strikt lokalen Prozesses und der Diagnose einer Infektion des aktiven kardialen Implantats analog der neuen Infektionskriterien aktiver kardialer Implantate der europäischen Gesellschaft für Kardiologie aus dem Jahr 2019 wurde zunächst die empirische medikamentöse antibiotische Therapie mit einem Cephalosporin begonnen und der Patient im Beisein der Angehörigen über die leitliniengerechte Therapie der Extraktion aufgeklärt.

Der Patient war nicht bereit, sich einer leitliniengerechten Therapie zu unterziehen. Die alternativ in Aussicht gestellte chronische Antibiotikatherapie in Kombination mit einem Unterdruckverband lehnte er ab.

Trotz historisch gesehen geringer Aussichten auf Erfolg [1] war der Patient einer Revision mit Resektion des Hautdefekts zugänglich. Auch der Entfernung der Vorhofsonde – sollte sich die Prozedur intraoperativ als unkompliziert durchführbar darstellen – stimmte er in letzter Konsequenz zu.

In der nun folgenden operativen Revision wurde ein elliptischer Hautschnitt um den Erosionsdefekt einschließlich der beiden Narbenlinien (Abb. 1) angesetzt. Anschließend wurde in der Tiefe der Schnittlinie mittels eines Niedrigtemperatur-Dissektionsinstruments (PEAK PlasmaBlade™, Medtronic, Minneapolis, MN, USA) sowohl der ICD-Generator als auch die ihn umgebende fibröse Aggregatstasche en-bloc mobilisiert. Vor Extraktion aus der fibrösen Tasche und Diskonnektion von den Sonden wurde der Situs mit einer antimikrobiellen Lösung auf Taurolidinbasis speziell für die Verwendung bei operativen Prozeduren aktiver kardialer Implantate zugelassen, gespült (TauroPace^TM^, TauroPharm, Bayern, Deutschland; [2, 3]). Nach Diskonnektion des Aggregats von den Sonden wurde das bereits kalzifizierte fibröse Gewebe, das die extravaskulären Segmente der Elektroden umgab, vollständig bis zum Eintrittspunkt der Sonden in das venöse Gefäß reseziert. Großes Augenmerk lag hier auf Unversehrtheit der Isolation der Sonden. Die Fixierhülsen um die Elektroden, die atriale Sonde und alle nichtresorbierbaren Nahtmaterialien wurden entfernt.

Nach Exzision aller fibrösen Kapselreste wurde der Situs erneut mit etwa 50 ml der taurolidinhaltigen Lösung gespült, wobei die zugänglichen Teile der verbleibenden Schockelektrode in der Lösung verweilten, während ein neuer Generator aus seiner sterilen Verpackung entnommen, mit der Lösung befeuchtet und in eine mit der Lösung getränkten Gaze eingewickelt wurde. Aufgrund der langen extravaskulären Anteile der Sonde wurde eine neue subkutane Tasche (anstelle der submuskulären) in kraniomedialer Position der alten Tasche vorbereitet und gründlich mit der taurolidinhaltigen Lösung gespült. Der extravaskuläre Anteil der Sonde und der Kontakt wurden mit taurolidinlösungsgetränkten Tupfern abgewischt, bevor dieser in den Port des neuen Aggregats gesteckt wurde. Der Drehmomentschlüssel wurde kurz in die taurolidinhaltige Lösung getaucht, bevor die Dichtung über der Fixierschraube durchstochen wurde. Das neue, mit der alten Schockelektrode verbundene Aggregat wurde in der neu präparierten Tasche positioniert. Weitere 10 ml der taurolidinhaltigen Lösung wurden vor Verschluss der Tasche in diese eingeträufelt. Nach Anlegen eines Wundverbands wurde ein Druckverband über der Tasche angebracht. Der Patient wurde unter fortgesetzter oral-antibiotischer Therapie mit Ampicillin/Sulbactam (2-mal/Tag; 7 Tage) in sein häusliches Umfeld entlassen.

**Figure 2:**
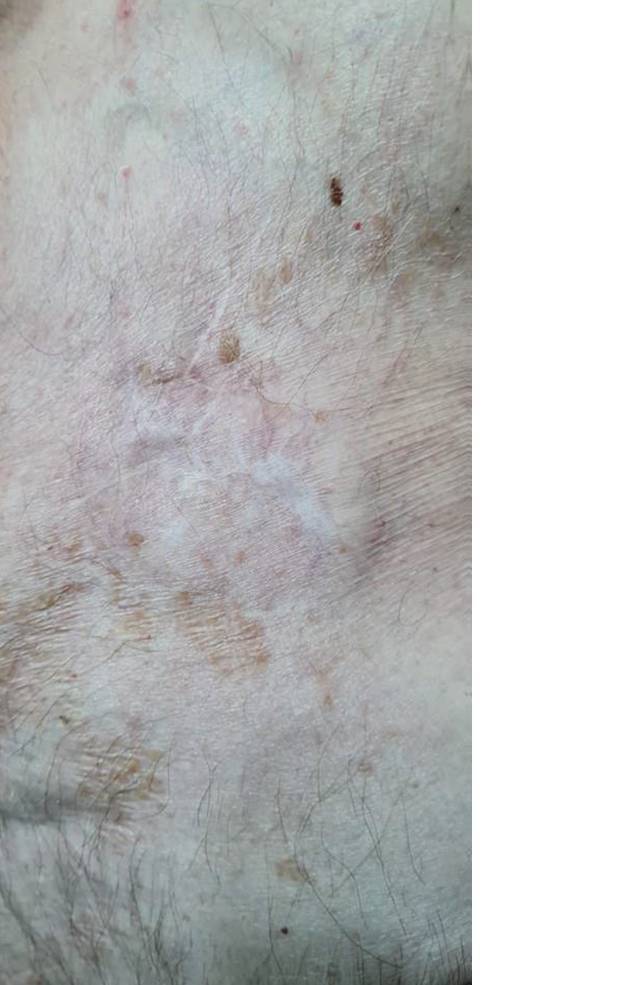

Der Patient erholte sich problemlos von dem Eingriff. Im Rahmen der Nachsorge nach 1, 4, 6, 9 und 15 Monaten postoperativ (Abb. 2) konnte ein Wiederauftreten der Infektion mithilfe von Blutuntersuchungen, Sonographie der Aggregattasche, transthorakaler und transösophagealer Echokardiographie ausgeschlossen werden.

## Diskussion

Jede Exposition von aktiven kardialen Implantaten, jede Fistel oder Hautadhäsion wird gemäß den neuen Kriterien als Infektion betrachtet [4]. Die Infektion ist gemäß den Leitlinien durch eine vollständige Entfernung aller Komponenten des aktiven kardialen Implantats zu behandeln [4, 5]. Bei gebrechlichen Patienten mit erheblichen Begleiterkrankungen oder begrenzter Lebenserwartung können konservative Maßnahmen wie regelmäßiger Verbandswechsel, Unterdruckwundverband und/oder chronische Antibiotikatherapie als Alternative in Betracht gezogen werden [4]. Unter bestimmten Umständen könnte das Aggregat gerettet werden – dies gilt auch für den Fall einer Kolonisierung des Aggregats [6], solange ein Wiederauftreten der Infektion ausbleibt.

Dieser Fall zeigt, dass es prinzipiell möglich ist, ein Aggregat durch ausgedehntes Débridement mit Entfernung aller potenziell betroffenen Gewebe, sorgfältiger Vermeidung einer Kreuzkontamination des neuen Operationsfeldes durch Verwendung von antimikrobiellen Lösungen für die In-vivo- und Ex-vivo-Desinfektion von Gewebe und Aggregat sowie die Schaffung einer neuen Tasche zur Aufnahme des Aggregats erfolgreich zu retten. Wenn die in diesem Fall erreichte mittelfristige klinische Infektionsfreiheit ohne vollständige Entfernung aller Komponenten bei weiteren Patienten erreicht werden kann, würde das beschriebene Verfahren das Spektrum der Maßnahmen erweitern, die für den Umgang mit infizierten aktiven kardialen Implantaten bei Patienten zur Verfügung stehen, die zu gebrechlich für die Anwendung von Standardverfahren sind und bei denen sich sowohl Patienten als auch Ärzte nicht auf einen konservativen Ansatz einigen können.

## Fazit für die Praxis

- Schrittmacherinfektionen stellen ein klinisches Dilemma dar, auch weil sie nicht immer auf den ersten Blick als Infektion zu identifizieren sind.
- Anhand dieser Kasuistik lässt sich die leitliniengerechte Therapie mit schneller und konsequenter Explantation aller Komponenten sowie eine potenzielle Vorgehensweise in Ermangelung einer leitliniengerechten Therapiemöglichkeit ableiten.
- Quintessenz bleibt, dass die Rhythmuschirurgie ein hohes Maß an Expertise, im Zweifel über den Bereich der Kardiologie oder Herz-Thorax-Chirurgie hinaus, erfordert.
